# Exploring the Ocular Surface Microbiome and Tear Proteome in Glaucoma

**DOI:** 10.3390/ijms25116257

**Published:** 2024-06-06

**Authors:** Livia Spörri, Anne-Christine Uldry, Marco Kreuzer, Elio L. Herzog, Martin S. Zinkernagel, Jan D. Unterlauft, Denise C. Zysset-Burri

**Affiliations:** 1 Department of Ophthalmology, Inselspital, Bern University Hospital, University of Bern, 3010 Bern, Switzerland; elio.herzog@extern.insel.ch (E.L.H.); martin.zinkernagel@insel.ch (M.S.Z.); jan.unterlauft@insel.ch (J.D.U.); denise.zysset@insel.ch (D.C.Z.-B.); 2 Department for BioMedical Research, University of Bern, 3008 Bern, Switzerland; anne-christine.uldry@unibe.ch; 3 Interfaculty Bioinformatics Unit and Swiss Institute of Bioinformatics, University of Bern, 3012 Bern, Switzerland; marco.kreuzer@unibe.ch; 4 Graduate School for Cellular and Biomedical Sciences, University of Bern, 3012 Bern, Switzerland

**Keywords:** liquid chromatography–tandem mass spectrometry, glaucoma, ocular surface microbiome, tear proteome, whole-metagenome shotgun sequencing

## Abstract

Although glaucoma is a leading cause of irreversible blindness worldwide, its pathogenesis is incompletely understood, and intraocular pressure (IOP) is the only modifiable risk factor to target the disease. Several associations between the gut microbiome and glaucoma, including the IOP, have been suggested. There is growing evidence that interactions between microbes on the ocular surface, termed the ocular surface microbiome (OSM), and tear proteins, collectively called the tear proteome, may also play a role in ocular diseases such as glaucoma. This study aimed to find characteristic features of the OSM and tear proteins in patients with glaucoma. The whole-metagenome shotgun sequencing of 32 conjunctival swabs identified Actinobacteria, Firmicutes, and Proteobacteria as the dominant phyla in the cohort. The species *Corynebacterium mastitidis* was only found in healthy controls, and their conjunctival microbiomes may be enriched in genes of the phospholipase pathway compared to glaucoma patients. Despite these minor differences in the OSM, patients showed an enrichment of many tear proteins associated with the immune system compared to controls. In contrast to the OSM, this emphasizes the role of the proteome, with a potential involvement of immunological processes in glaucoma. These findings may contribute to the design of new therapeutic approaches targeting glaucoma and other associated diseases.

## 1. Introduction

Glaucoma is a worldwide leading cause of irreversible vision loss. It is characterized by the gradual degeneration of retinal ganglion cells and their axons [1,2], associated with narrowing of the visual field [3]. In 2013, the global occurrence of glaucoma among individuals aged 40 to 80 years was approximately 3.5%. The total number of glaucoma patients was nearly 80 million in 2020, and it is projected to surpass 100 million by the year 2040 [4,5].

Glaucoma can be divided into several subtypes, including primary open-angle glaucoma (POAG), primary angle-closure glaucoma (PACG), and secondary glaucoma. In POAG, serious damage may be caused to the optic nerve and the trabecular meshwork, which is the main pathway for drainage of aqueous humor and whose dysfunction leads to intraocular pressure (IOP) elevation [6]. The most common form of secondary glaucoma is pseudoexfoliation glaucoma (PEXG), caused by the deposition of fibrillar protein aggregates (PEX fibrils) on the anterior and posterior eye tissues [7].

IOP is the only modifiable risk factor that can influence the progression of glaucoma [8]. Treatment options to reduce IOP includes topical and oral therapies, laser treatments targeting the trabecular meshwork or ciliary body, and incisional surgery [2,5,9]. In addition to IOP elevation, the risk factors for glaucoma include advancing age, gender, diet, obesity, depression, and anxiety [5]. Since there are associations between the gut microbiome and these risk factors [10,11,12,13,14,15], the gut microbiome has gained much attention in the pathophysiology of glaucoma. Several studies identified significant changes in the microbial composition of glaucoma patients compared to healthy controls, with a potential influence of neuroinflammation and autoimmunity [16]. 

However, while there are associations between gut microbiome dysbiosis [17,18,19] and the development of glaucoma [5], as well as other diseases, the role of the ocular surface microbiome (OSM) in glaucoma has not been explored so far. The OSM refers to the collective community of microorganisms and their genomes on the surface of the eye, including bacteria, viruses, and fungi [20,21,22,23,24,25,26,27]. The investigation of the OSM in this study stems from a gap in prior research, particularly the Human Microbiome Project, which primarily focused on characterizing microbiomes across various body sites but excluded the eye [28]. Unlike other areas explored, such as the skin, gastrointestinal tract, and oral cavity, the OSM has been relatively unexplored. Modern sequencing technologies are employed to gain a comprehensive understanding of the OSM, including its interactions with antimicrobial proteins in tear fluid [22] and involvement in eye diseases such as dry eye disease [29]. To gain insights into the complex interplay between the OSM, tear proteome, immune system processes, and glaucoma, the OSM and tear fluid of 16 glaucoma patients, compared to 16 healthy controls, were explored in this study. Furthermore, since the previous research has shown alterations in bacterial composition and diversity due to various types of eye drops, such as antibacterial agents and sodium hyaluronate [30,31], the influence of IOP-reducing eye drops on the OSM and its potential implications for ocular health were assessed.

Prebiotics, probiotics, fecal transplantation, and more recently postbiotics have been suggested as interventions to modulate the gut microbiome, thereby ameliorating associated conditions [32]. This study enables the identification of potential targets for glaucoma treatment by microbiome-altering approaches and aid in the development of personalized therapeutic strategies in the future [33].

## 2. Results 

### 2.1. Demographic Data

A total of 32 conjunctival swabs, as well as the correspondent tear fluids, were collected from 16 glaucoma patients and 16 healthy controls. While the two groups did not show differences in the sex ratio (Table 1), there was a significant difference in age between the groups (*p* = 0.0046, Welch’s *t* test). Since previous studies have shown an age-dependence for both the OSM [34,35] and the tear proteome [36], we considered this difference in a Multivariate Association with Linear Models (MaAsLin2) analysis [37] (see Section 2.2 and Section 4.3) and screened for age-independent protein expressions (see Section 2.3 and Section 4.4). In the glaucoma group, there were eight patients with POAG and eight patients with PEXG. A subgroup analysis was performed for the OSM characterization (see Section 2.2). 

The ocular hypotensive therapy for glaucoma frequently incorporates active pharmaceutical ingredients such as alpha-agonists, beta-blockers, carbonic anhydrase inhibitors, or prostaglandin analogs to reduce IOP. In our cohort of 16 patients, the therapeutic regimens for glaucoma management revealed a distribution of ocular hypotensive agents as follows: prostaglandin analogs (14 patients), carbonic anhydrase inhibitors (13 patients), beta-blockers (15 patients) and alpha-agonists (6 patients; Table 2). Notably, the majority of patients were subjected to multiple agents concurrently, indicative of prevalent combination therapies in the management of glaucoma.

### 2.2. Characterization of the Ocular Surface Microbiome in Glaucoma Patients

In total, 1.83 billion 150 bp paired-end reads with an average insert size of 350 bp were generated, with an average of 78.21 ± 22.46 (s.d.) million reads per sample. As expected and described in previous studies, most of these reads were of human origin due to the low abundance of the OSM [38]. After trimming and host-filtering, we kept about 331 million high-quality, non-human reads with an average of 6.94 ± 4.59 (s.d.) million per sample. After removing duplicated reads using Unique Molecular Identifiers (UMIs), we had a total of 4.27 million deduplicated reads for further analysis, with an average of 0.17 ± 0.14 (s.d.) million per sample. Since sequencing libraries from negative controls failed quality and quantity controls for sequencing, we concluded that contaminations were not present in a significant concentration.

**Figure 1:**
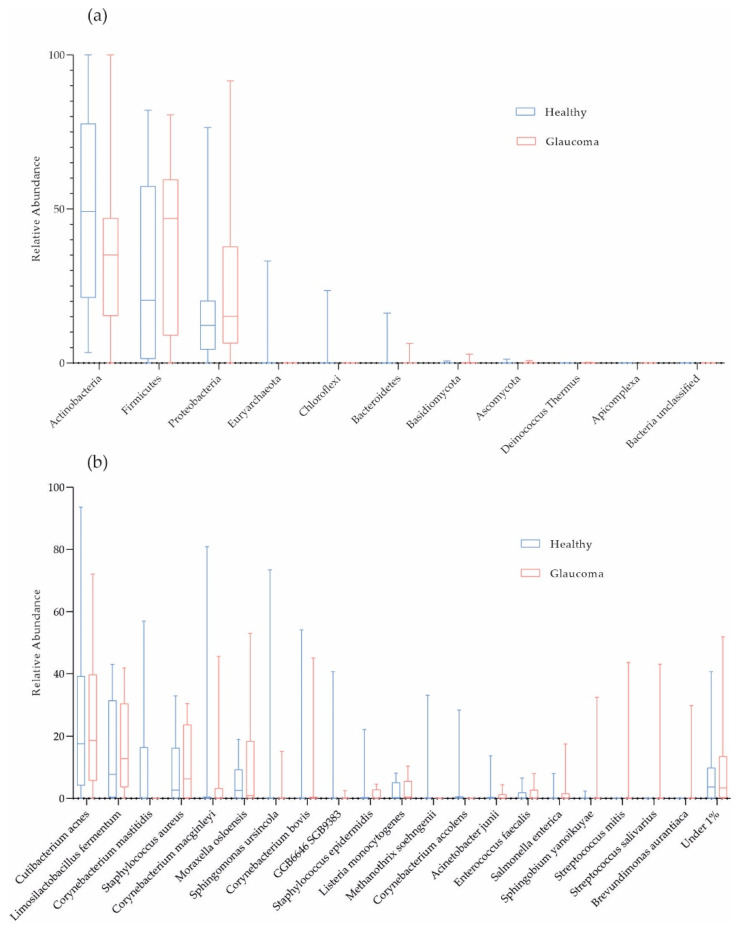
Taxonomic characterization of ocular surface microbiome. Box plots for relative abundances at phylum (**a**) and species level (**b**) are shown (red, glaucoma, n = 16; blue, healthy controls, n = 16).

On average, 98.72% of the host-filtered reads were of bacterial origin. Consistent with previous studies, the phyla Actinobacteria (41.50%), Firmicutes (33.51%), and Proteobacteria (22.10%) dominated OSM composition in the cohort (Figure 1a). *Cutibacterium acnes* (24.17%), *Limosilactobacillus fermentum* (15.42%), and *Staphylococcus aureus* (9.84%; mean values) were the most abundant species in the cohort (Figure 1b). In Figure 1b, on average, we filtered 9.19% and 6.71% of the data for patients and controls, respectively, because these taxonomic units were present at less than 1% (low-abundant species termed “Under 1%”).

**Figure 2:**
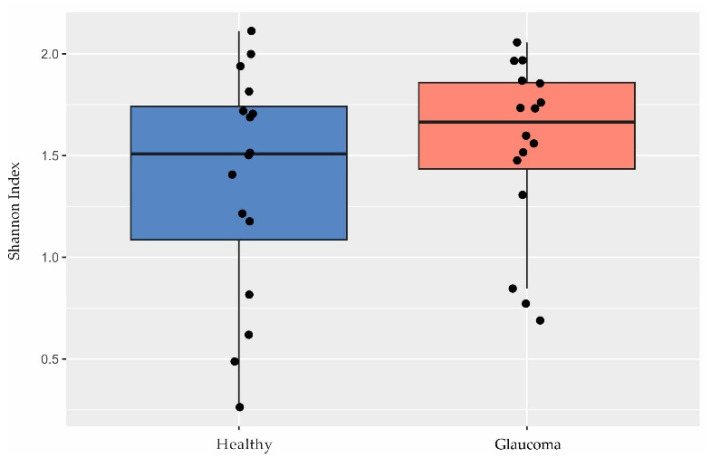
Diversity in the ocular surface microbiome. There were no differences in Shannon index observed between glaucoma patients (n = 16) and controls (n = 16; *p* = 0.35, Welch’s *t* test).

Although other studies have shown a lower microbial diversity in glaucoma [39,40,41], we found no differences in diversity between patients and controls based on the Shannon index (*p* = 0.35, Welch’s *t* test; Figure 2).

**Figure 3:**
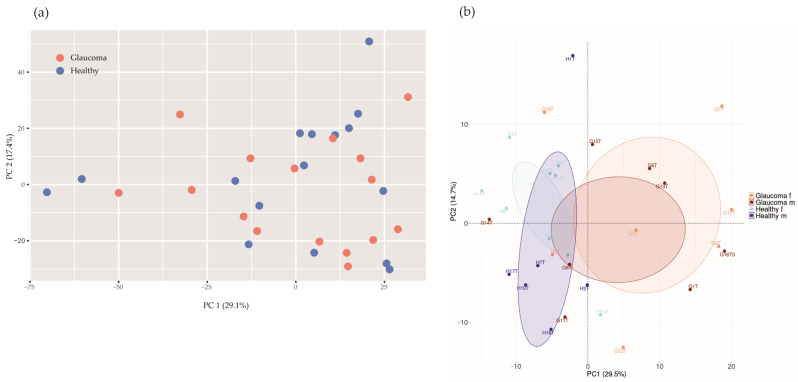
Principal component analysis (PCA) of microbial abundances (**a**) and protein intensities (**b**). (**a**) PCA of microbial abundances did not separate glaucoma patients (red, n = 16) from healthy controls (blue, n = 16; *p* = 0.51, PERMANOVA, *n* repeat = 1000). (**b**) Log2−transformed protein group intensities before imputation were used for analysis. PCA separates glaucoma patients (n = 16) from controls (n = 16; *p* = 0.0060, PERMANOVA, *n* repeat = 1000). Orange, female glaucoma patients (n = 7); red, male glaucoma patients (n = 9); light blue, healthy control females (n = 10); blue, healthy control males (n = 6).

If applying a principal component analysis (PCA) with health status as the grouping variable, the glaucoma group would not separate from healthy controls based on differences in microbial abundances (*p* = 0.51, PERMANOVA, *n* repeat = 1000; Figure 3a). 

To further examine taxonomic and functional features of the OSM in glaucoma, we used MaAsLin2 to investigate whether the relative abundances of microbial taxa and pathways were associated with glaucoma and/or demographic data.

**Figure 4:**
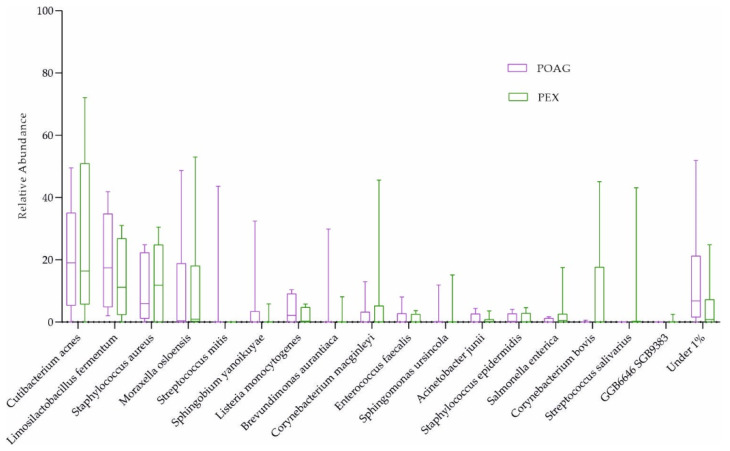
The ocular surface microbiome of POAG and PEXG patients. Box plots for relative abundances of all species are shown. PEXG, pseudoexfoliation glaucoma (n = 8); POAG, primary open-angle glaucoma (n = 8).

*Corynebacterium mastitidis* was absent in all glaucoma patients, but it was present in 7 out of 16 controls (Figure 1b and Table 3). However, there were no significant differences in microbial abundances between the subgroups (combination of four active ingredients of IOP-lowering eye drops including alpha-agonists, versus three active ingredients, versus no eye drops; Table 2; and POAG versus PEXG patients; Figure 4). For functional features, the ocular microbiomes of controls seem to be enriched in genes of the phospholipase pathway (q = 0.14).

### 2.3. Functional Annotation of the Tear Proteome in Glaucoma

We quantified 2250 human tear proteins. Applying a PCA based on log2-transformed protein group intensities, there are two clusters according to study groups (glaucoma versus controls; Figure 3b), suggesting a glaucoma-specific protein expression in tear fluids. 

**Figure 5:**
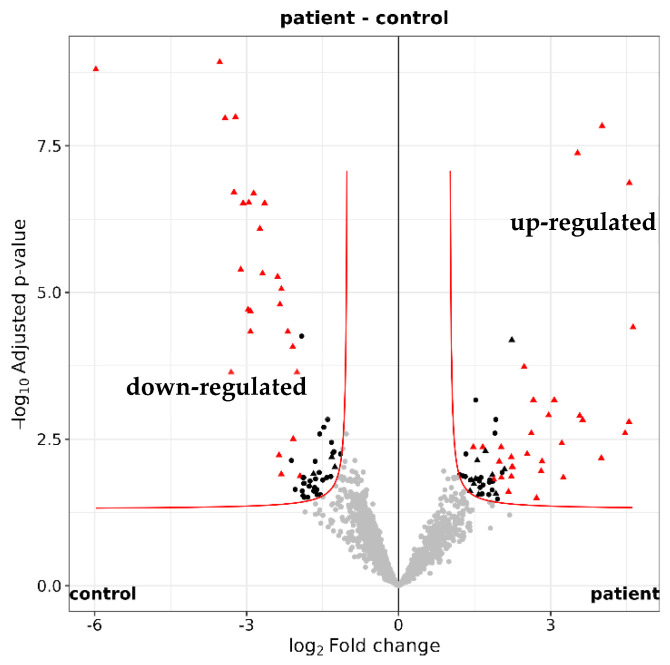
Quantification of the tear proteome. Volcano plot illustrating the 2250 identifed tear proteins. A total of 123 proteins with a maximum adjusted *p* value of 0.05 were either up-regulated (log2 fold change ≥ 1) or down-regulated (log2 fold change ≤ −1) in glaucoma patients (n = 16) versus healthy controls (n = 16).

Among the 2250 identified tear proteins, 123 proteins were different in quantity between glaucoma patients and controls (according to imputed iMAX LFQ (label-free quantification) values, maximum adjusted *p*-value < 0.05 and fold change ≥2 or ≤−2; Figure 5 and Supplementary Table S1). Using STRING version 12.0, a functional enrichment analysis showed that many of the differentially expressed proteins are involved in biological processes associated with the immune system (Table 4). 

## 3. Discussion

### 3.1. Implications of the Ocular Surface Microbiome in Glaucoma

Glaucoma, a prevalent and irreversible vision-impairing condition, is characterized by the gradual decline of retinal ganglion cells and their axons [1,2], resulting in narrowing of the visual field [3]. Over the past decade, there has been an increasing focus on exploring the connection between microbiota and glaucoma [5,6,42,43]. Microbiota imbalance (dysbiosis) triggered by various glaucoma risk factors, including aging [44], obesity [45], and depression [46], can lead to disruptions in metabolic, immune, and inflammatory processes. These disruptions are crucial mechanisms contributing to the development of the disease [5,43,45,47,48]. This study aimed to investigate the OSM and tear proteome in glaucoma patients compared to sex-matched healthy controls. The OSM, comprising microorganisms and their genomes on the eye’s surface, has been relatively unexplored compared to other body sites [20,28]. 

Several studies corroborate the prevalence of our top three bacterial species at phylum level, validating their prominence in ocular microbiomes [22,29,38,49,50,51,52,53]. Glaucoma patients and controls shared Actinobacteria, Proteobacteria, and Firmicutes as the predominant phyla in their OSMs, with Actinobacteria being the most abundant in our cohort. Their dominance underscores their potential significance in shaping the OSM. On species level, *Corynebacterium mastitidis* was only found in 7 out of 16 healthy controls but not in any of the glaucoma patients. *C. mastitidis* is a bacterium known for its commensal presence on mucous membranes, particularly on the ocular surface. It has been associated with promoting beneficial local immune responses [54]. The absence of *C. mastitidis* in glaucoma patients raises the possibility that its presence may play a stabilizing role in ocular health, suggesting a potential link between its absence and the disease. In an experimental model simulating ocular surface disease in mice, researchers found a persistent colonization of the ocular mucosa by *C. mastitidis*. They showed a causal relationship between the presence of *C. mastitidis* in the eyes and the promotion of beneficial local immune responses [55]. In another mouse model, *C. mastitidis* has been associated with the development of inflammation and abscesses, playing a role in protecting the eyes by releasing antimicrobials. It was demonstrated that *C. mastitidis* is found in both humans and mice [54]. St Leger et al. further showed that *C. mastitidis* triggers γδ T cells to produce interleukin 17, leading to the release of these antimicrobial molecules into tears. This protective mechanism provides a defense against invasive *Candida albicans* and *Pseudomonas aeruginosa* infections. These findings underscore the existence of true commensalism at the ocular surface, emphasizing the crucial role of local γδ T cell responses and suggesting potential broader implications for ocular surface diseases [56]. 

Functional analysis of the OSM identified a phospholipase pathway that may be increased in healthy controls compared to patients. The relationship between phospholipids and glaucoma has been explored, highlighting phospholipase A2 (PLA2) as a contributor to neuronal cell death, particularly in the trabecular meshwork (TM) of glaucoma patients. The suppression of lipid mediators generated by PLA2 activity presents a potential therapeutic target for treating glaucoma. However, the study emphasizes the need for caution in developing safe and isoform-specific PLA2-targeting drugs, to minimize the associated risks and side effects [57]. The potental inverse up-regulation in our study needs further investigation.

### 3.2. The Influence of Intraocular Pressur-Lowering Eye Drops on the Ocular Microbiome

In addition to exploring microbial composition and function in glaucoma, our study investigated the influence of IOP-lowering eye drops on the OSM. Although previous studies have demonstrated alterations in ocular microbiota following the use of various eye drops [30,31], we did not detect any influence of eye drop composition on the OSM. 

A randomized trial comparing Carboxymethylcellulose to Polyethylene Glycol artificial tears found specific changes at genus level, including an increase in *Bacteroidota* and *Actinobacteriota* with Carboxymethylcellulose treatment [58]. However, overall diversity of the OSM was not significantly altered by these interventions. Limited research exists on the impact of IOP-lowering eye drops on the OSM, emphasizing the importance of further investigation. One study on the prolonged use of prostaglandin analogs in glaucoma treatment found a stable OSM among participants but lacked a healthy control group for direct comparison [59]. Similarly, a study comparing eyelid microbiomes in glaucoma patients found no association between microbiome composition and the use of preservative-containing eyedrops [60]. Furthermore, a study assessing the impact of topical antiglaucomatous eye drops on conjunctival flora in glaucoma patients and healthy controls revealed a significant increase in bacterial isolates in glaucoma patients, particularly *Staphylococcus hominis* and *Candida* spp. [61]. However, comparing conjunctival bacterial cultures in patients undergoing glaucoma and cataract surgery showed no significant difference in positive bacterial growth between the two groups, suggesting that glaucoma medications or their preservatives may not significantly alter the OSM [62]. In a research study that examined individuals with asymmetrical glaucoma and treated only one eye with preserved topical glaucoma therapy containing benzalkonium chloride, it was discovered that the OSM in the treated eye displayed a wide variety of Gram-negative bacteria. This differed significantly from the predominantly Gram-positive microbes found in healthy controls, suggesting a possible connection between microbial alterations and inflammation of the ocular surface, as well as changes in tear film and protein synthesis [63]. Overall, while various eye drops, including those used for glaucoma treatment, can impact the OSM, the extent of these changes and their clinical implications warrant further investigation. Therefore, continued research is essential to elucidate the potential systemic effects of ocular treatments and to optimize care for patients with ocular conditions.

### 3.3. Implications of the Tear Proteome and the Immune System in Glaucoma

Apart from the exclusive presence of *C. mastitidis* in controls and its potental association with ocular homeostasis, the OSMs from patients and controls seem to be comparable. This suggests a minor role of the OSM in glaucoma and is in contrast to many tear proteins with different abundances in glaucoma. The functional analysis of these tear proteins revealed an up-regulation of immune-related pathways in glaucoma patients compared to controls. In contrast to the OSM, this outcome suggests an important role of the tear proteome associated with the immune system in glaucoma. 

While the innate immune system has long been linked to glaucoma through glial cells and oxidative stress, the findings support the role of the adaptive immune system [64]. Studies have shown that CD4+ T cells can facilitate RGC death [65], and an imbalance in Treg cells/T17 cells has been observed in experimental autoimmune optic neuritis, a condition sharing similarities with glaucoma [66]. Studies indicate a correlation between immune potency and resistance to RGC death, suggesting immune dysfunction as a potential condition for glaucoma development [67]. 

Despite being primarily associated with elevated IOP [68], the molecular mechanisms behind RGC death in glaucoma are complex and unclear [69]. Various factors contribute to RGC degeneration [70], including caspase activation [71,72], apoptosis [73], oxidative stress [74], ischemia [75], hypoxia, epigenetic changes [76], inflammatory cytokines [77], and neurotrophic factor deprivation [78]. Emerging evidence suggests the involvement of T cells in glaucoma pathology, with studies showing T cell infiltration in response to an elevated IOP and continued RGC degeneration [79].

In summary, subsequent investigations have identified autoantibodies targeting retinal and optic nerve proteins [70] in the serum [80], retina [81], and aqueous humor [82] of individuals with glaucoma. Additionally, research has indicated the existence of autoantibodies targeting heat shock proteins in individuals with glaucoma [83], which are molecular chaperones that can be up-regulated during stress [84] and potentially induce molecular mimicry, leading to the development of autoantibodies against host proteins [85]. Increased levels of specific heat shock proteins have been observed in glaucoma, with some potentially contributing to retinal ganglion cell death [86]. Administering heat shock proteins to rats through immunization has been demonstrated to cause glaucomatous damage in some studies [87]. Conversely, alternative research has indicated that the induction of heat shock proteins can confer neuroprotection for retinal ganglion cells [88,89,90]. In general, the involvement of autoantibodies and heat shock proteins in glaucoma is still not clearly understood.

### 3.4. Conclusions

In conclusion, the exploration of the OSM and tear proteome in glaucoma patients has provided insights into potential drivers of the disease and opened up promising avenues for targeted therapies. Previous studies proposed the use of animal models to examine changes in the microbiome pre- and post-glaucoma induction, aiming to determine whether these changes are a direct result of glaucoma or secondary to treatments involving preservative-laden eye drops. While existing studies have used mouse models to induce glaucoma [91,92,93,94], research specifically focusing on the OSM in these models has been limited.

Contrary to our initial expectations, our findings suggest that the OSM plays a minor role compared to the tear proteome in influencing the immunological processes associated with glaucoma. The significant enrichment of immune-related pathways observed in the tear proteome of glaucoma patients highlights the complexity of ocular surface homeostasis and its implications for disease pathogenesis. These results underscore the potential of the tear proteome as a rich source for innovative diagnostic and therapeutic strategies in glaucoma management.

By focusing on the tear proteome and its interaction with immune responses, personalized interventions tailored to individual patient profiles may offer new opportunities for improved clinical outcomes. Future research should continue to explore these interactions in greater depth, paving the way for the development of new treatment modalities that are more effective and less invasive than current options. This comprehensive approach is crucial for enhancing our understanding of glaucoma mechanisms and ultimately improving patient care.

## 4. Materials and Methods

### 4.1. Study Design and Recruitment

This study was approved by the Ethics Committee of the Canton of Bern (ClinicalTrials.gov: NCT04656197). The procedures followed the tenets of the Declaration of Helsinki and the International Ethical Guidelines for Biomedical Research involving Human Subjects (Council for International Organizations of Medical Sciences). After receiving oral and written information, all participants gave written informed consent before study enrollment. Participants (n = 32) were consecutively recruited from the Department of Ophthalmology of the University Hospital Bern (Inselspital), Switzerland. Patients with a history of recent (last 3 months) ocular surgery and systemic or topical antibiotics within the last 3 months were excluded. Participants fulfilling all of the following inclusion criteria were eligible for the study. The participants were willing to sign informed consent, were 50 years of age or older, and underwent glaucoma surgery within 24 h (glaucoma group). They were not eligible for the study if they were not willing or able to sign informed consent, were younger than 50 years, had a recent (3 months) history of use of systemic and/or topical antibiotics or ocular surgery, or a history of glaucoma (healthy control group). 

### 4.2. Sample Collection 

After verifying that no exclusion criteria were met and written informed consent was given, the tear fluid was collected by Schirmer’s type 1 tear test. A standard filter strip (Haag-Streit AG, Köniz, Bern, Switzerland) was inserted into the lower conjunctival bag of both eyes. After 5 min, the strip was removed and immediately processed for tear fluid extraction as described in [22]. The extracted tear fluid was stored at −80 °C until further analysis by nano-liquid chromatography–tandem mass spectrometry (nLC-MS/MS; Section 4.4). 

A total of 32 conjunctival swabs using flocked nylon swabs (FLOQSwabs #518CS01, Copan, Brescia, Italy) from 16 patients and 16 controls were collected [38]. A local anesthetic (Tetracaine 1%, Théa, Schaffhausen, Switzerland) was applied. In controls, we performed a pooled swab from both eyes, while in the glaucoma group, we performed the conjunctival swab on the eye with glaucoma and a planned surgery within the next day. For negative controls, flocked nylon swabs with one drop of Tetracaine 1% (n = 2) were processed in parallel to conjunctival swabs.

### 4.3. Metagenomic DNA Sequencing and Analysis

Conjunctival swabs, as well as negative controls, were processed, and DNA was isolated on the same day using the QIAamp DNA Microbiome Kit (51704) from QIAGEN (Hilden, Germany). DNA was stored at −20 °C until further analysis. The DNA was then used to prepare sequencing libraries with the NEBNext Ultra II Preparation kit (SKU E7645L). The dual indexed libraries were sequenced on an Illumina NovaSeq 6000 sequencer for 150 paired-end cycles. Sequencing was performed at the Next-Generation Sequencing Platform of the University of Bern, Switzerland.

The resulting DNA sequences contain a Unique Molecular Identifier (UMI; [95]). The UMIs were first extracted using UMI-tools (v. 1.1.4). The reads were then quality-filtered using fastp (v. 0.20.1; [96]. Then, the reads were mapped to the human genome reference GRCh38 in order to remove the host DNA. Unmapped reads were extracted using SAMtools (v.1.10). The host-filtered reads were then mapped to the ChocoPhlAn reference database version mpa_vOct22_CHOCOPhlAnSGB_202212 using bowtie2 (v. 2.3.4.1; [97]). The resulting SAM alignment file was converted to the BAM format and then sorted and indexed using SAMtools (v. 1.10; [98]). The resulting file was then used to deduplicate the reads in the alignment based on the UMI and mapping location, using UMI-tools (v.1.1.4). The deduplicated BAM file was then used as input for the MetaPhlAn4 (v. 4.0.4; [99,100]) pipeline and the extracted deduplicated fastq files for the HUMAnN3 (v. 3.8) [101] pipeline.

In order to provide a global analysis of microbial abundances by PCA, the function adonis2 of the vegan R package and ggplot2 version 3.5.0 were applied. A PCA was performed using scaled values on the relative abundances of microbial species. A permutation multivariate analysis of variance (PERMANOVA) applying the R package vegan 2.6–4 was assessed with 1000 permutations to provide a *p* value for separation.

The Shannon index was calculated using vegan 2.6–4 and plotted by ggplot2 version 3.5.0. Associations of microbial and functional features of the microbiome with glaucoma or IOP-lowering eye drops, respectively, were identified by the MaAsLin2 R package, applying the default settings and using “groups” (glaucoma versus healthy controls, POAG versus PEXG patients, combination of four active ingredients of IOP-lowering eye drops including alpha-agonists, versus three active ingredients, versus no eye drops) as fixed and “age” as a random effect. 

### 4.4. Tear Fluid Processing and Analysis [102,103,104,105,106,107,108,109,110,111]

For tear fluid collection, the tear fluid-soaked Schirmer strip was put into a 0.5 mL Protein LoBind tube (Eppendorf AG, Hamburg, Germany, 0030108094) punctured at the bottom with a cannula. This tube was placed into a larger 2 mL Protein LoBind tube (Eppendorf AG, Hamburg, Germany, 0030108132) and centrifuged at maximum speed (10,000 rpm) for 1 min. This procedure was initially described by Posa et al. [112] and allows the extraction of tear fluid. Tear fluid was stored at −80 °C until further analysis by nLC-MS/MS. A total of 2 µL human tear fluid was diluted with 8 µL 8M urea/100 mM Tris, reduced for 30 min at 37 °C with 0.1M DTT/100 mM Tris, alkylated for 30 min in darkness with 0.5M IAA/100 mM Tris at 37 °C, and quenched with DTT. The sample was filled up to 20 µL with 6× Laemmli buffer and heated to 95 °C for 5 min prior to loading on a 12.5% SDS-PAGE gel. The gel was run to about 1.5 cm length, stained with Coomassie blue, and each lane was cut into five bands equal in size. Gel bands were cut into small cubes, which were transferred to 1.5 mL polypropylene reaction vials and wetted with 100 µL ethanol for storage at 4 °C until digestion.

For nLC-MS/MS, the 36 samples were randomized in order to minimize the memory and matrix effect of the injections. Proteins were in-gel digested as described by Gunasekera et al. [113]. An aliquot of 5 µL from each digest was analyzed by an nLC-MS/MS instrument consisting of an EASY-nLC 1000 chromatograph coupled to a QExactive HF mass spectrometer (ThermoFisher Scientific). Peptides were trapped on an Acclaim C18 PepMap100 pre-column (5 μm, 100 Å, 300 μm × 5 mm, ThermoFisher Scientific, Reinach, Switzerland) and separated by backflush on a C18 column (3 μm, 100 Å, 75 μm × 15 cm, Nikkyo Technos, Tokyo, Japan) by applying a 40 min gradient of 5% acetonitrile to 40% in water, 0.1% formic acid, at a flow rate of 350 nL/min. Peptides of *m*/*z* 400–1400 were detected at a resolution of 60,000 (at *m*/*z* 250) with an automatic gain control (AGC) target of 1E06 and maximum ion injection time of 50 ms. A top-fifteen data-dependent method for precursor ion fragmentation was applied with the following settings: precursor ion isolation width of 1.6 *m*/*z*, resolution 15,000, AGC of 1 × 10^5^, maximum ion time of 110 ms, charge inclusion of 2+ to 7+ ions, peptide match on, and dynamic exclusion for 20 s, respectively.

The mass spectrometry data were searched and quantified with FragPipe [fragpipe] version 20.0, using MSFragger [msfragger] version 3.8. The database used was the Human swissprot [uniprot] database (release April 2023) with isoforms and concatenated to common contaminants. The precursor and fragment tolerance were set, respectively, to 15 and 20 ppm. The search enzyme was set to trypsin, with a maximum number of allowed missed cleavages of 3. Carbamidomethylation on cysteine was set as a fixed modification; methionine oxidation, glutamine and asparagine deamidation, and protein N-terminal acetylation were given as variable modifications. The minimum of matched fragments was set to 5. Validation (Philosopher [philosopher] version 5.0.0) was performed with the Peptide Prophet option, and a protein false discovery rate of 0.01. Quantification was conducted with IonQuant [ionquant] version 1.9.8 (outcome named FragI) and Label Free Quantification (outcome named MaxLFQ). A match between runs was enabled, for a maximum of 10 top runs.

Proteins with less than 2 identified peptides were first removed from the list. After filtering off contaminants, proteins with less than 8 identifications in either control or patients, and less than 9 or 8 identifications in, respectively, the female or male groups, were discarded. MaxLFQ values were re-normalized by vsn [vsn] on this set of proteins. Missing values were imputed at protein level (iMaxLFQ) by drawing random values from a Gaussian distribution of width 0.3 × sample standard deviation and centered at the sample distribution mean minus 2.5 × sample standard deviation (protein level). Differential expression tests were performed with the moderated *t* test of the R limma package [ebayes], and the Benjamini and Hochberg [BH] method was further applied to correct for multiple testing. The criterion for statistically significant differential expression was that the maximum adjusted *p*-value for large fold changes was 0.05, and that this maximum decreased asymptotically to 0 as the log2 fold change of 1 was approached (with a curve parameter of 0.1 times the overall standard deviation; Figure 5). Proteins consistently significantly differentially expressed through 20 protein imputation cycles were subsequently flagged [transport]. 

Following the submission of a list containing all proteins identified in our samples with the fold change (iMaxLFQ log2FC patient–control, biological process, and iMaxLFQ EB patient–control, biological process) to STRING version 12.0 for analysis, we obtained a compilation of all biological processes analyzable in our samples. Subsequently, these processes were ranked based on the false discovery rate, which is a measure for the accuracy of the results. In order to provide a global analysis of protein abundances, the PCA was performed as described above. 

## Figures and Tables

**Table 1:** Demographic information of study participants.

	Total	Female	Male	Age Mean	SD	Variance (Age)	Chi-Squared Test (Sex)
Healthy	16	10	6	68.1	7.3	56.3	0.54
Glaucoma	16	7	9	76.5	7.7	63.2	

**Table 2:** Topical intraocular pressure-lowering active ingredients of all 16 glaucoma patients.

Patient	Prostaglandin Analogs	Carbonic Anhydrase	Beta-Blockers	Alpha-Agonists
1	x	x	x	
2	x	x	x	x
3	x		x	
4	x	x	x	x
5	x		x	
6	x	x	x	
7	x	x	x	x
8	x	x	x	x
9	x	x	x	
10	x	x	x	x
11	x	x	x	x
12	x	x	x	
13		x	x	
14				
15	x	x	x	
16	x	x	x	

**Table 3:** Number of *Corynebacterium mastitidis*-positive samples. Contingency table, *p* = 0.0068 (two-tailed Fisher’s exact test).

*C. Mastitidis*	Positive	Negative
glaucoma	0	16
controls	7	9

**Table 4:** Functional annotation of tear proteins in glaucoma. Functional enrichment analysis of differentially expressed proteins between glaucoma patients and controls (based on iMAX LFQ values). The top first GO (gene ontology) terms are shown (sorted by false discovery rate).

GO Term ID	Biological Process	Genes Mapped	Enrichment Score	False Discovery Rate
GO:0006952	Defense response	153	1.62573	1.28 × 10^−12^
GO:0006959	Humoral immune response	59	2.35722	1.28 × 10^−12^
GO:0006955	Immune response	141	1.49942	5.32 × 10^−12^
GO:0098542	Defense response to other organism	123	1.61702	3.21 × 10^−10^
GO:0042742	Defense response to bacterium	45	2.36236	3.48 × 10^−8^
GO:0045087	Innate immune response	98	1.52697	2.90 × 10^−17^
GO:0006954	Inflammatory response	55	1.65598	6.60 × 10^−7^
GO:0006956	Complement activation	27	1.24414	1.04× 10^−6^
GO:0002682	Regulation of immune system process	133	2.08046	1.27 × 10^−6^
GO:0019730	Antimicrobial humoral response	30	2.82318	1.27 × 10^−12^

## Data Availability

The datasets supporting the conclusions of this article are available in the European Nucleotide Archive under the accession number PRJEB74248.

## Supplementary Materials

The following supporting information can be downloaded at: https://www.mdpi.com/article/10.3390/ijms25116257/s1.
